# Brain-Computer Interface Controlled Cyborg: Establishing a Functional Information Transfer Pathway from Human Brain to Cockroach Brain

**DOI:** 10.1371/journal.pone.0150667

**Published:** 2016-03-16

**Authors:** Guangye Li, Dingguo Zhang

**Affiliations:** State Key Laboratory of Mechanical Systems and Vibrations, Institute of Robotics, Shanghai Jiao Tong University, Shanghai, China; Universität Bielefeld, GERMANY

## Abstract

An all-chain-wireless brain-to-brain system (BTBS), which enabled motion control of a cyborg cockroach via human brain, was developed in this work. Steady-state visual evoked potential (SSVEP) based brain-computer interface (BCI) was used in this system for recognizing human motion intention and an optimization algorithm was proposed in SSVEP to improve online performance of the BCI. The cyborg cockroach was developed by surgically integrating a portable microstimulator that could generate invasive electrical nerve stimulation. Through Bluetooth communication, specific electrical pulse trains could be triggered from the microstimulator by BCI commands and were sent through the antenna nerve to stimulate the brain of cockroach. Serial experiments were designed and conducted to test overall performance of the BTBS with six human subjects and three cockroaches. The experimental results showed that the online classification accuracy of three-mode BCI increased from 72.86% to 78.56% by 5.70% using the optimization algorithm and the mean response accuracy of the cyborgs using this system reached 89.5%. Moreover, the results also showed that the cyborg could be navigated by the human brain to complete walking along an S-shape track with the success rate of about 20%, suggesting the proposed BTBS established a feasible functional information transfer pathway from the human brain to the cockroach brain.

## Introduction

The movie “Avatar” shows the dream of direct brain-to-brain control between different individuals, which has inspired researchers to develop physical communication connections between different brains. Some pilot studies have moved a step forward toward this fantastic dream through developing a brain-to-brain system (BTBS). Pais-Vieira *et al*. [1] developed a BTBS between two rats based on invasive technologies (neuronal ensemble recordings and intracortical microstimulation), where the “decoder” rat could follow the same sensorimotor task as the “encoder” rat with the system. Yoo *et al*. [2] established functional interface between brains of human and rat based on noninvasive technologies, electroencephalography (EEG) and focused ultrasound (FUS), where the rat’s tail could be controlled by a human’s intention. Rao *et al*. [3] realized a BTBS between two persons based on noninvasive technologies, EEG and transcranial magnetic stimulation (TMS), in which the receiver’s (a subject) finger motion could be triggered by a sender (the other subject). The previous studies are amazing, but the function of such BTBS is very limited, i.e. only a simple motion or action can be accomplished. This work aims to develop a versatile multi-functional BTBS between humans and cockroaches.

A typical BTBS should consist of two key parts: brain-computer interfaces (BCIs) used for getting information from one brain and neuromodulation technologies used for sending information to another brain. BCI is a well-known technology that helps people communicate with external worlds through measuring and translating brain signals without involving muscular movements or peripheral nervous system [4]. Using BCIs, humans have already could control external devices such as robotic arm, wheel chair, quadcopter and so on [5–7]. In general, there are two categories of BCIs, i.e. invasive and non-invasive BCIs. Non-invasive BCIs include EEG, near-infrared spectroscopy (NIRS), and functional magnetic resonance imaging (fMRI) etc. While, electrocorticography (ECoG) and intracortical recordings belong to the scope of invasive BCIs [8, 9]. Among the available BCIs, EEG should be the most practical and convenient method at present, and steady-state visual evoked potential (SSVEP) is one of the most reliable, versatile and robust EEG paradigms, which is thus adopted in our study [10].

SSVEP is an EEG response from the visual cortex generated by repetitive visual stimuli when subjects gaze at flickering source [11]. SSVEP has high signal-to-noise ratio (SNR) and information transfer rate (ITR), and needs short training time. These favorable features of SSVEP are desired for the high-performance BTBS developed in this study. Even though SSVEP can be elicited by visual stimulation with a wide range of frequencies from 1–100 Hz, the better SSVEP performance is achieved under visual stimulation with lower frequencies range of 6–15 Hz in general [12, 13].

Neuromodulation, as an intervention technology used to modulate the nervous system function, has been applied to the brain, spinal cord, peripheral nerves, autonomic nerves and other nervous organs [14]. Among the variety of technologies used for neuromodulation (mechanically, chemically and electrically, etc.), electrical stimulation-based neuromodulation is frequently introduced when developing live cyborgs or biobots. In fact, a variety of cyborgs (a cyborg is an organism with both biological and electronic parts) have been successfully developed via applying electrical stimulation to specific muscles or nerves in recent years, such as rats, moths, and beetles [15–19]. To build up a cyborg that could be remotely controlled by a human brain, cockroach is a preferable choice for robust control performance and good surgical implementability. It’s known that cockroaches rely on antennas heavily for navigation during walking. The antenna is a multi-functional and important sensory organ that can generate tactile, thermal, humidity and olfactory sense. When sending specific micro-electrical pulse trains through the antenna nerve, stimulation information will be encoded as sensory information by the antennal neurons and sent to the brain, which will activate the descending mechanosensory interneurons (DMIs) (interneurons with the largest caliber axons descending to thoracic levels from the brain) [20, 21]. Subsequently, the thoracic motor centers will be activated, and as a result, the evasive behaviors such as turning will be elicited [21–24]. If an electrical pulse train is applied to the nerves of right (left) antenna, a left (right) -direction turn will be triggered from the cockroach. By inserting a tiny electrode into antenna and providing electrical stimulation, a functional neural-machine interface between external device and cockroach brain can be developed.

Based on the present technologies of BCI and neuromulation, we find it is possible to realize communication and virtual signal transfer from one brain to another one with a functional BTBS. This interesting concept is explored in this study and we aim to control cyborg cockroaches to accomplish path-tracking tasks with human brain signals for the first time.

## Materials and Methods

### Overview of BTBS

**Fig 1:**
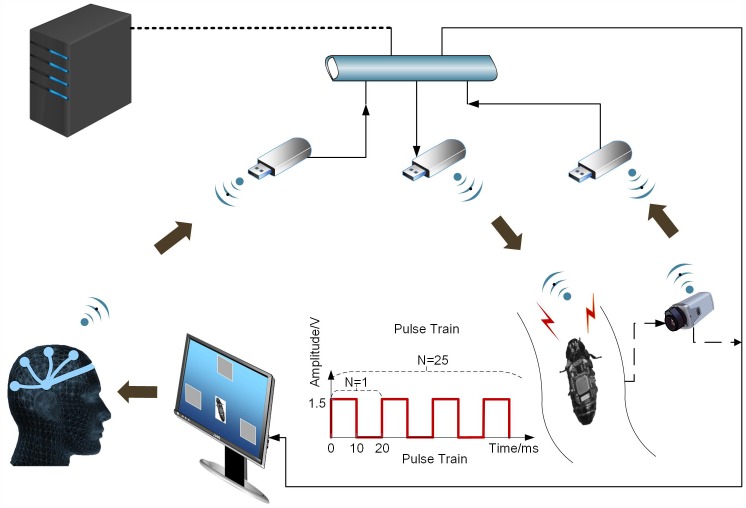
System Overview. Entire system consists of an SSVEP-based BCI, a cyborg cockroach, and a communication sub-system. The communication sub-system transfers the real-time BCI commands from the host computer to the cyborg cockroach. The controller wirelessly steers the cyborg cockroach using his/her brain signals from the LCD screen.

The BTBS that can navigate a cockroach by human brain was developed, and the framework of the entire system is shown in Fig 1. SSVEP-based BCI was utilized to decode the sender’s thoughts, and a cyborg cockroach was developed to be the receiver and execute the expected motion tasks from the sender. In addition, an imperative wireless communicative sub-system between these two parts was established to transfer the sender’s command from the host computer to the receiver (cyborg cockroach). We also used a wireless video capture module in the SSVEP program to visually obtain the real-time response of the receiver from an LCD screen, therefore, a closed-loop control system was established. The system was implemented with the following configurations.

A USB dongle, which had a system-on-chip CC2541 (Texas Instrument Inc.), was used and plugged in the host computer to pair with the microstimulator wirelessly. Besides, two custom softwares were developed for this system and run in the host computer. SSVEP program (SSVEP Cyborg, v1.0, SJTU) was written in Visual C++ to be used as an integration platform for the whole system (acquiring realtime brain signal, processing and generating classification outputs), while another custom software (BLE Control Panel, v1.0, SJTU) was programmed in Visual C# to realize the communication between the microstimulator and SSVEP program as well as adjustment of stimulus parameters for the electronic backpack. A large number of offline tests were conducted in pilot study for the purpose of getting proper stimulation parameters, and reaction from the cyborg cockroach was evaluated by its turning angle and reaction duration after stimulation. Finally, a 1.5 V, 50 Hz, 50% duty cycle and 500 ms monopole square pulse train was used because such neurostimulation configuration was effective and optimal to the navigation of the cyborgs.

### Implementation of SSVEP-based BCI

**Fig 2:**
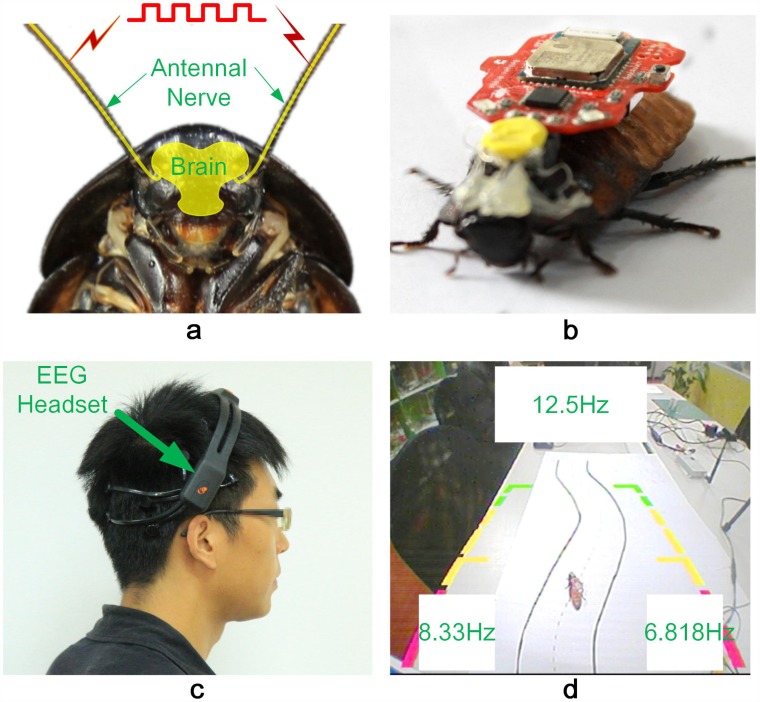
Experimental set-up. a. Nerve system of a cockroach and electrodes implantation. b. A cyborg: a cockroach with a microstimulator on the back. c. A human subject wearing an EEG headset. d. Snapshot of the SSVEP user interface used in online control session.

EEG data were recorded with the Emotiv EPOC headset (Emotiv Company) reversely wore on the head of subjects (Fig 2c). This portable brain headset had 14 EEG channels and used 2 scalp reference electrodes. All electrode impedances were kept below 10 kΩ, four standard electrodes in the EEG headset were used and were positioned over the visual cortex (locations PO3, PO4, O1, O2 according to the international extended 10/20 system, two CMS/DRL reference electrodes were placed on C5/C6). The raw EEG was down-sampled at 128 Hz and the band pass was set to 0.16–43 Hz after applying a notch filter at 50 and 60 Hz automatically on the incoming signal. The data were wirelessly transmitted to the host computer through a Bluetooth USB chip operating in 2.4 GHz by using an Emotiv Testbench software (Emotiv Systems). This EEG headset had been validated on its performance and had been put into scientific use in the previous studies [25–28].

Human subjects sit about 0.7 m in front of a 44.5 cm × 38.5 cm LCD screen (refreshing rate 75 Hz) on which visual stimuli were provided in the form of three flashing blocks. The flickering frequency of each block was set as 12.5 Hz, 8.33 Hz, 6.818 Hz (Fig 2d), denoting the rest, left-turn and right-turn control commands respectively, and size of the three blocks was 13.6 cm (length) × 7.0 cm (height) / (upper block), 6.9 cm (length) × 7.4 cm (height) / (left and right block). The background of the SSVEP on the LCD screen was set as the real-time images of the experimental environment captured by a wireless video capture system, which consisted of a portable camera (ND-101, NBC Inc.), a wireless video transmitter and receiver module (FOX-800A, Botong Inc.), and a wireless USB video capture card (UV200, 10moons Inc.). The camera had a CMOS image sensor with 512×582 pixel and was fixed in a 50cm-high position on the experiment desk.

**Fig 3:**
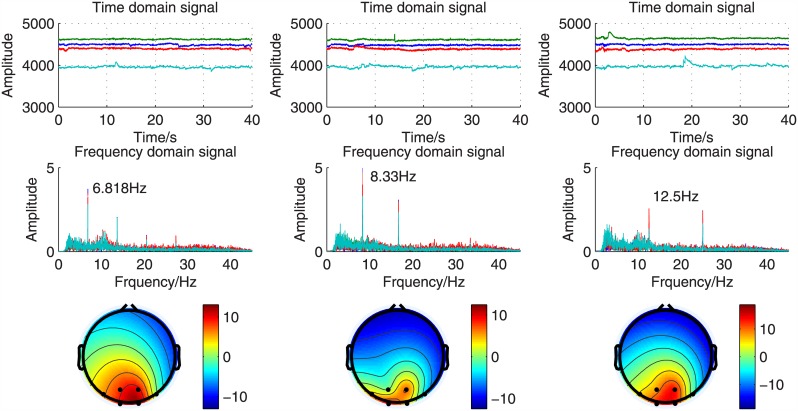
Plots of EEG signals in time domain, frequency domain, and spatial domain. The first row of the graphs are 40-second EEG signal taken from 4 channels when a subject facing visual stimuli with three different frequencies separately. Relevant spectrum is presented in the second row, and the third row is the power distribution on brain map.

Before the start of experiments, time-domain EEG signals, which were taken from above 4 channels by gazing each single flicker separately for 40 s, were plotted in Fig 3. Clear and strong response distributed on each frequency could be found from Fig 3, demonstrating the three frequencies used in this study were highly effective.

### Implementation of Cyborg Cockroach

The Madagascar hissing cockroach (Gromphadorhina portentosa) was selected as the model insect for the reason that it was strong, robust. Moreover, it has relatively larger size (∼ 50–80 mm) and slower walking speed [22]. These cockroaches were purchased from Chinese market.

To develop a cyborg cockroach, a microstimulator (Roboroach, v1.1b, Backyard Brains Inc.) was used. Three silver wires (0.06 mm bare/0.08 mm coated) were used as electrodes to transfer the stimulation pulses. Strands of 60 mm wires were taken and the insulation was removed at either ends for about 5 mm. One end of the electrodes was soldered to the pins of a 2.54-mm (spacing distance) header male connector, and the other end was inserted 3–5 mm into the cockroach antenna after partially removing the flagellum of both the antennae to 8–10 mm. For effective insertion and minimally disturb the insects, anesthetization was applied to the cockroach through cold-treatment in ice for about 10 minutes. The male connector was firstly placed on the cockroach pronotum with super glue after drying the cockroach, and then placed the ground electrode in the first segment of the thorax though an incised hole (left and right side could be used either, we stabbed the hole in right side of the thorax in this study). Super glue was used to seal off all above points after successful insertion. Finally, the microstimulator (Weight, 4.72 g; Size (mm), 34.00/L * 25.4/W * 6.9/H) could be fixed on the back of the cockroach by plugging it in the male connector and a living cyborg could be seen in Fig 2b.

### Algorithms for BCI Classification

EEG signals (4 channels) were acquired and primarily processed with an online 4-order Butterworth 2 Hz high-pass filter in the Matlab (Mathworks, Natick, MA), filtering the frequency band to 2–43 Hz. Feature extraction was firstly applied on the filtered signal and then a linear discriminant analysis (LDA) classifier was introduced to process the EEG signals.

#### Feature Extraction

Based on the previous study of SSVEP [13], principle frequency component and harmonic wave components of the signal were used for feature extraction with the way of projecting the signal to sine and cosine space (Inner Product, Eqs (1)-(6)). The discrete formulations were given below:

$$a_{1}^{ij}\left(n\right)=\left\langle S_{i}\left(m\Delta T\right)W\left(m\Delta T-ns\right),\cos\left(2\pi f_{j}m\Delta T\right)\right\rangle$$ (1)

$$b_{1}^{ij}\left(n\right)=\left\langle S_{i}\left(m\Delta T\right)W\left(m\Delta T-ns\right),\sin\left(2\pi f_{j}m\Delta T\right)\right\rangle$$ (2)

$$a_{2}^{ij}\left(n\right)=\left\langle S_{i}\left(m\Delta T\right)W\left(m\Delta T-ns\right),\cos\left(4\pi f_{j}m\Delta T\right)\right\rangle$$ (3)

$$b_{2}^{ij}\left(n\right)=\left\langle S_{i}\left(m\Delta T\right)W\left(m\Delta T-ns\right),\sin\left(4\pi f_{j}m\Delta T\right)\right\rangle$$ (4)

$$A_{ij}\left(n\right)=\sqrt{{\left|a_{1}^{ij}\left(n\right)\right|}^{2}+{\left|b_{1}^{ij}\left(n\right)\right|}^{2}}$$ (5)

$$B_{ij}\left(n\right)=\sqrt{{\left|a_{2}^{ij}\left(n\right)\right|}^{2}+{\left|b_{2}^{ij}\left(n\right)\right|}^{2}}$$ (6)

where *S*_*i*_ is the *i* th channel signal with *i* = 1…*N*; *m* is the *m* th signal point with *m* = 1…*L* (*L* is the total signal length); *W*(*mΔT* − *ns*) is the rectangle windows function at the center *ns*, *ΔT* is the sampling interval, *ΔT* = 1/*fs*; *f*_*j*_ is the *j* th stimuli frequency with *j* = 1…*M*; *A*_*ij*_(*n*) and *B*_*ij*_(*n*) is the *n* th principle frequency and harmonic wave feature of *i* th channel signal in *j* th stimuli frequency respectively; windows length used is 1 second in this study, *s* is the sliding step of the signal window, *s* = 0.125 second.

Thus, a feature vector **F** ((2×N×M)×1) could be extracted in following form:

$$F={\left(A_{11},...A_{ij},...A_{NM},B_{11},...B_{ij}...B_{NM}\right)}^{T};i=1...N,j=1...M$$ (7)

#### Classification

An LDA classifier was applied to make quick classification using the 120-second training set after feature extraction. The parameters of the LDA classifier can be derived by Fisher method which searches an optimal projection direction that maximizes difference between the two classes while minimizes difference within the class [29].

The multi-dimension data is then discriminated in the linear formulation as

$$y=w^{T}F+w_{0}$$ (8)

where **w** is the optimal projection direction.

Since we use 3 classes, the classification result *C* in each signal window length is derived:

$$C=\underset{i}{\arg\max}\left\{\sum_{j=1,j\neq i}^{3}y_{ij},i=1,2,3\right\}$$ (9)

Define *y*^*ij*^ = 1, if *y*^*ij*^>0; else *y*^*ij*^ = 0, where *y*^*ij*^ is the function of *i* (Eq (8)).

### Optimization Algorithm for SSVEP-based BCI

Considering that a higher online classification accuracy from the SSVEP was essential to this online control system, however, the discrete results generated directly from the LDA classifier generally carried random errors in a much high possibility, therefore, an optimization algorithm aimed to improve the online recognition accuracy of SSVEP-based BCI was designed and then verified in the following experiment (Phase I). By constructing a buffer pool for the original discrete results from the LDA classifier, the algorithm could obviously enhance the fault-tolerant capacity and at the same time increase the rate of correct outputs. The algorithm was described below:

Setting a variable *Z*_*i*_ (*i* = 1, 2, 3), which represents the dynamic accumulation value belongs to *i* th class of SSVEP. Initial value of *Z*_*i*_ is set to 0. With the running of SSVEP classifier, a classification result *C* (Eq (9)) will be generated internally in each sliding step (0.125s), causing simultaneously a change of *Z*_*i*_ when following each cycle of below arithmetic rule,

1. For *i* = 1, 2, 3;
2. if *C* = *i*, *Z*_*i*_ = *Z*_*i*_+*V*_*positive*_;
3. if *Z*_*i*_ ≥ *V*_*negative*_, *Z*_*i*_ = *Z*_*i*_ − *V*_*negative*_;
4. if *Z*_*i*_ ≥ *V*_*threshold*_, the *i* will be the final control command of SSVEP that will send to the cyborg, after a command is triggered, *Z*_*i*_ = *Z*_*i*_ − *V*_*minus*_;
5. go back to step 1.

where a series of *V* values are pre-set performance-adjustment parameters of the SSVEP program. Among these parameters, *V*_*positive*_ and *V*_*negative*_ determine the speed of triggering an output, *V*_*threshold*_ controls the precision level of generating a final result, and *V*_*minus*_ is used to adjust the time interval between two continuous outputs. Different combinations of the *V* values are tested and configured finally as (*V*_*positive*_ = 20, *V*_*negative*_ = 10, *V*_*threshold*_ = 40, *V*_*minus*_ = 20) in this study to achieve favorable performance of the SSVEP program (response fast and high accuracy, see Results Phase I).

### Human Subjects, Insects and Ethics Statement

Six healthy subjects were recruited and participated in the following experiments. All participants were fully informed about the experimental procedure and the written informed consents were obtained from all participants before experiments. During the online control experimental phase, three cyborg cockroaches were made and used. The information of human subjects and cockroaches was given in Table 1. All animal procedures were performed in accordance with the National Research Council’s Guide for the Care and Use of Laboratory Animals and the whole study was approved by the Ethics Committee of Shanghai Jiao Tong University, China. Besides, the individual in this manuscript has given written informed consent (as outlined in PLOS consent form) to publish these case details.

**Table 1:** Information of human participants/cockroaches.

	Age(years)	Gender	Experiment
Subject_1	28	M	Phase I &II
Subject_2	24	M	Phase I &II
Subject_3	24	M	Phase I &II
Subject_4	22	M	Phase I
Subject_5	25	M	Phase I
Subject_6	25	M	Phase I
	Age(years)	Length(cm) * Weight(g)	Experiment
Cockroach_1	1.5	6.1 * 7.74	Phase II
Cockroach_2	1.5	5.8 * 6.95	Phase II
Cockroach_3	1.5	5.8 * 8.23	Phase II

### Experimental Protocol

Experiments were divided into two phases in this study, phase I was set to validate the effect of applying the optimization algorithm in the SSVEP program, and phase II was to explore the online control performance of current control system.

#### Phase I: Optimization Algorithm Verification

**Fig 4:**
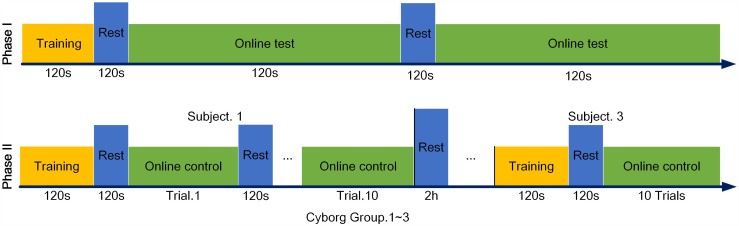
Experimental paradigm for Phase I and Phase II.

Only BCI part was established in this phase for the reason that optimization process could work internally in the SSVEP program without really navigating a cyborg. A virtual rotated ball, which acted as both a guidance of the subject’s visual attention and a mimic representation of the subject’s willingness, randomly appeared near one of three flickering blocks for 5 s. Experiment was conduct according to the protocols in Fig 4. In the end of experiment, the acquired EEG signals were comparatively analyzed in Matlab between running and without running the optimization algorithm.

#### Phase II: System Performance Evaluation

Cyborg cockroaches were placed at a distance of about 1.5 m from the host computer on a white sheet with a black-boundary S-shape track. Size of the S-shape track was 750 mm (vertical length) / 790 mm (stretched length) × 135 mm (width). Full shot of the cyborg cockroach and the experimental platform were wirelessly projected into the LCD screen as background of the SSVEP program (Fig 2d). Three cyborg cockroaches (Table 1) were made in this experiment.

For the purpose of making better internal comparative analysis on performance-related parameters from both the cyborg and the human aspect as well as giving a general evaluation on the entire system, three of six human subjects (Table 1) were randomly selected as controllers and the three subjects took online control of each cyborg in sequence to walk along the S-shape track after the cyborg started to move forward from the start point of the track with a certain speed range (1.5–5.5 cm/s). Therefore, three experimental groups were divided respectively based on the order of each cyborg.

Ten online-control trials were conducted for each subject and cyborg. A 2-minute rest was guaranteed between trials to minimize the effects of fatigue from both human and insects. To make sure the cyborg cockroach recover its sensitivity to stimuli, a rest of at least 2 hours was given between two experiments (Fig 4). Trajectories of the cyborg were captured by a digital video camera (HDR-XR350E, SONY Inc.).

Besides, some other parameters related to cyborg and microstimulator were pre-measured through offline tests in this phase for the goal of making precise control. The time lag counted from the sending out of a command via the SSVEP program to the corresponding response of the electronic backpack, was measured with the custom software written in Visual C# (cf. Materials and Methods) and the result was 130.8±6.6 ms under a distance of about 1.5 m from the host computer to the electronic backpack. The duration time counted from reception of a stimulation command to completion of reaction on the neural stimulation just received by the cyborg, was 642±134 ms when the given length of stimulation was 500 ms. So the time length from sending out a command by the SSVEP program to the completion of reaction by a cyborg cockroach was about 772 ms. Therefore, we set the minimal stimulation interval as 1000 ms in the SSVEP software to avoid disordering the cyborg.

## Results

### Phase I: Optimization Algorithm Verification

**Fig 5:**
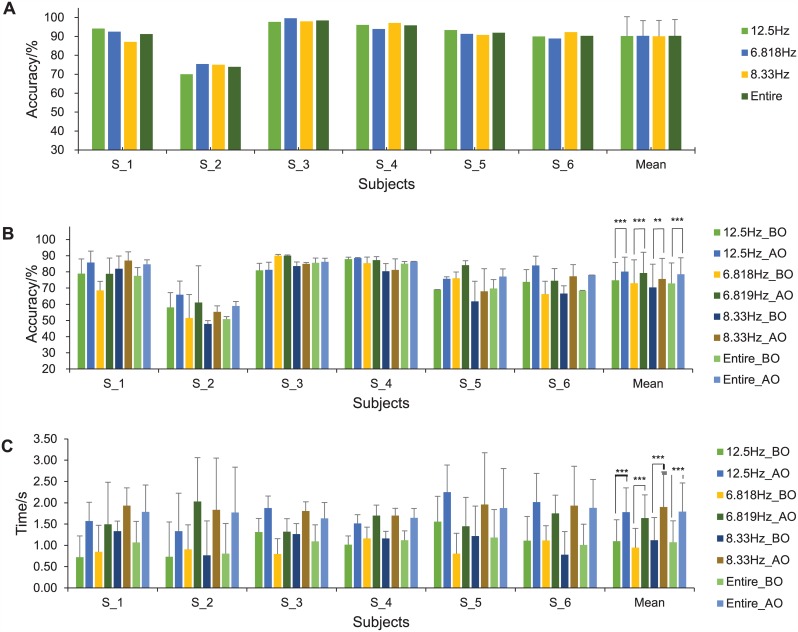
Experiment results before optimization (BO) and after optimization (AO). A. Classification accuracy (CA) in training phase. B. Online classification accuracy for all sub-classes and entire experiment. C. Recognition time in transient state for all sub-classes and entire experiment in online test session. (*** indicates the *P*<0.001, ** indicates the *P*<0.01.)

Fig 5 presented the accuracy and time information before optimization (BO) and after optimization (AO) through the experimental Phase I in detail, including three sub-classes and the mean value of entire session. The classification accuracy (CA) was firstly obtained (10-fold cross-validation) during training session before the start of online optimization, and then the value of recognition time (Time lag for classifying the first correct result in transition state) was calculated during transition state (The beginning of shift on flickering frequency). Finally, using the classifier generated in the training session, we computed the online classification accuracy before and after applying the optimization algorithm in the online test session (Accuracy obtained using the optimization algorithm was computed by: Accuracy = NC/NT (NC (NT); Number of correct results (total results) in each 120-s test session generated by the introduced algorithm)).

Mean value in Fig 5 also showed an overall statistical changes of SSVEP outputs. As a result of the optimization, a 5.70% increase from 72.86±13.03% to 78.56±10.45% in online classification accuracy of entire session could be achieved (*t* = -5.279, *P* = 0.0003, Paired T-test). Besides, the online classification accuracy of all the sub-classes increased significantly as well (*P*<0.005), 5.42%, 6.32%, 5.26% increment for 12.5 Hz, 6.818 Hz, 8.33 Hz respectively (Fig 5). Furthermore, the value (online classification accuracy) in entire session increased by 8.08% from 83.34±15.59% to 91.42±12.79% (*t* = -5.697, *P* = 0.0001, Paired T-test) after removing signals of the transition state. Meanwhile, the recognition time of the entire session from the SSVEP program turned significantly a little bit longer from 1.07±0.13 s in transient state and 0.125 s (minimum) in steady state to 1.79±0.11 s (*t* = -11.517, *P*<0.0001, Paired T-test) and 0.25 s (minimum). The time delay was then cross-evaluated through recent published researches [1–3], and results demonstrated response of the program using this algorithm was still comparatively quick enough. Therefore, the optimization process, which contributed to the improvement of online classification rate under an acceptable time delay, was verified as a superior strategy in online control and hence was introduced into the following experiments in Phase II.

### Phase II: System Performance Evaluation

**Fig 6:**
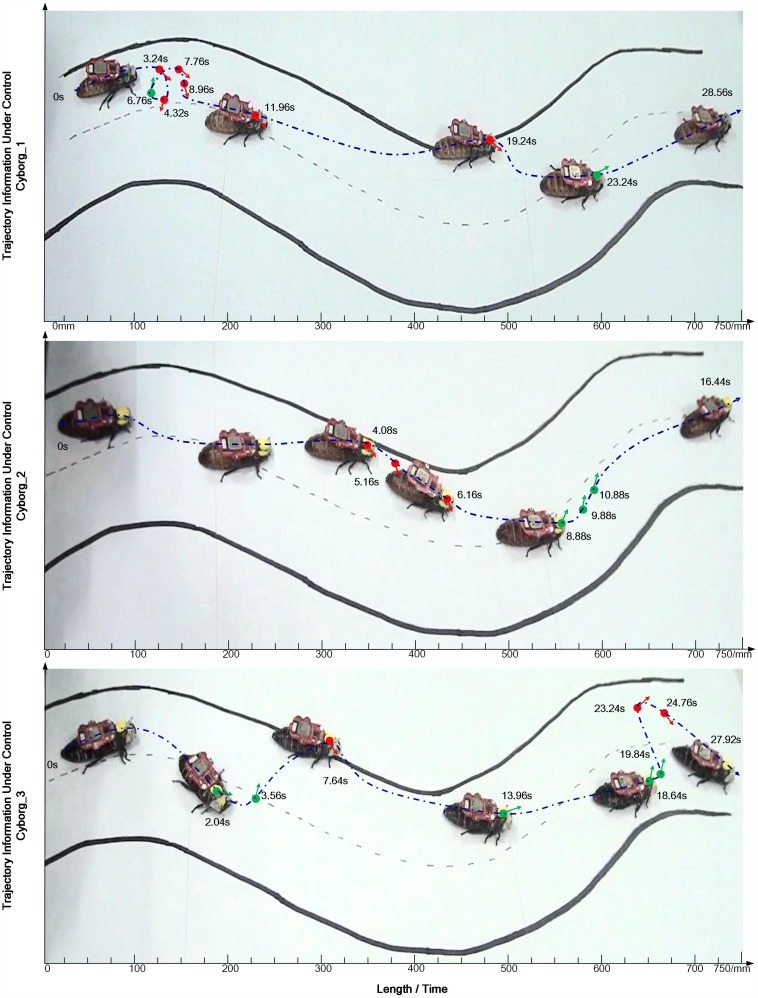
Trajectories of three cyborgs when walking along the S track under wireless BCI control of subjects. A green dot indicates a left-turn command sent from the subject, a red dot indicates a right-turn command. Corresponding time information in each trial is labelled as well.

Experiments were then conducted following the experiment paradigm described in Phase II. Fig 6 illustrated one track of each cyborg cockroach during online control, as can be seen from the figure, real-time human intention from the BCI were transferred to navigate the cyborg cockroach successfully, indicating as well the successful establishment of a practicable functional BTBS.

**Fig 7:**
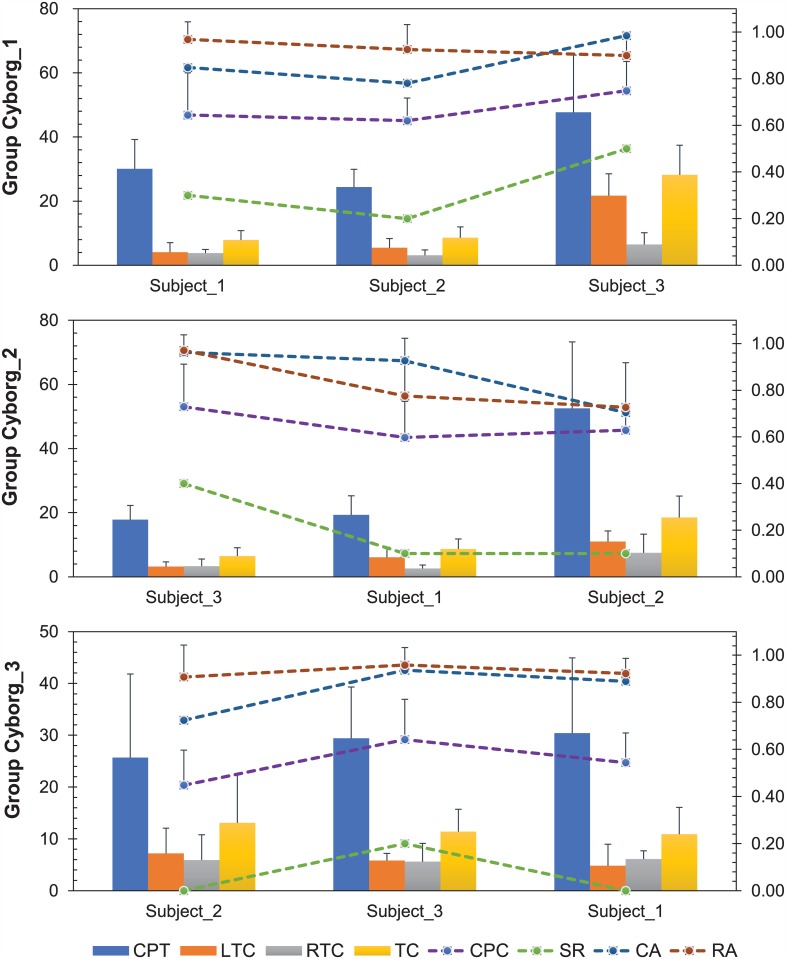
Results acquired in experimental phase II from three groups. CPT was measured in seconds, LTC, RTC and TC were counted in number, their value were shown on primary axis located in the left edge of above figure; RA from each cyborg, CPC, SR and CA were presented in decimal number in secondary axis located in the right edge of above figure. (Notes: Positive Standard Deviation are presented only.)

The time that a cyborg took to complete walking along the track was recorded in each trial and called completion time (CPT) in short. Since one class (12.5Hz) we set in the SSVEP program was resting state and would cause no reaction from the cyborg, only the number of left-turn commands (LTC) and number of right-turn commands (RTC) sent out by the SSVEP in each trial were imperative to be counted so that we can generally measure the effects of micro electrical stimulation on the cyborg. Number of turning commands (TC), which was the sum of left-turn commands and right-turn commands, was listed as well (Fig 7 & Table 2).

**Table 2:** Summary of system-performance-related parameters.

Group	CPT(s)	TC(LTC/RTC)	CA	RA	CPC	SR
Cyborg_1	33.9±15.4	15.0±11.1(10.6±9.1/4.4±2.8)	87.1±10.4%	93.1±9.5%	0.653±0.170	33.3±15.3%
Cyborg_2	22.9±20.4	11.2±6.9(6.8±4.2/4.5±4.1)	86.4±14.0%	82.4±20.8%	0.652±0.153	20.0±17.3%
Cyborg_3	28.5±13.5	11.8±6.5(5.9±3.8/5.9±3.5)	85.0±11.2%	92.9± 9.6%	0.544±0.165	6.7±11.5%
**Mean**	**30.8±16.7**	**12.7±8.5(7.8±6.5/4.9±3.5**)	**86.0±10.4%**	**89.5±15%**	**0.616±0.169**	**20.0±17.3%**

Abbreviations List: CPT, completion time; LTC/RTC/TC, number of left/right-turn commands/turning commands(LTC+RTC); CPC, control performance coefficient; SR, success rate; CA, classification accuracy; RA, response accuracy.

In addition, control performance coefficient (CPC) was proposed as one of indications of control performance based on following rules, and four labels based on the theory of receiver operating characteristics (ROC) were defined to cover all the possibilities that would occur during the actual situation [30]. The occurrences of true positives (TP: an expected command is sent to the cyborg and the cyborg turns in an expected direction), false positives (FP: an expected command is sent to the cyborg but the cyborg turns in an unexpected direction), false negatives (FN: an unexpected command is sent to the cyborg but the cyborg turns in an expected direction), true negatives (TN: an unexpected command is sent to the cyborg and the cyborg turns in an unexpected direction as well) were recorded. Response accuracy (RA) of the cyborg was computed using the equation RA = (TP+TN)/(P+N), where P = TP+FN, N = FP+TN.

Besides, a scoring strategy was adopted to quantify the CPC, which defined that a TP scored 3, a FP scored 2, a FN scored 1 and a TN scored 0, the related score was represented by a variable *X*. During the controlling process, the cyborg might go out of the boundary of the S-shape track, taking this into consideration, we then introduced an accuracy factor *α*, where *α* was 1 when the cyborg was walking inside the boundary, otherwise *α* was 0.5. The CPC was defined as CPC = 1/3*n* $\sum_{i=1}^{n}$ *X*_*i*_ *α*_*i*_, where *X*_*i*_ was the *i* th performance scores of the stimulation command, *α*_*i*_ was the *i* th accuracy factor, *n* was the TC in a trial.

Furthermore, successful rate (SR) was computed after each 10-trial session, where completing walking along the S-shape track without going outside of the boundary was counted as a successful trial.

Relevant parameters of each cyborg group (cf.Materials and Methods) were attained and shown in Fig 7 in detail. We found the parameter value varied among subjects and groups, however, some general features could be discovered. The subject (subject_3) had the best control performance (SR as high as 50%) on three cyborgs where his CA was the highest, and the subject (subject_2) had the lowest SR among three groups where his CA was the lowest as well. The Pearson correlation test was then conducted to analysis if there existed certain correlation between each pair of parameters. The correlation coefficient between SR and CA was *ρ* = 0.6059, where the value was *ρ* = 0.3798 between SR and RA, and *ρ* = 0.6227 between SR and the product of CA and RA (CA*RA), the results indicated that SR did have certain correlation with CA (from the controller) and RA (from the cockroach), but fully correlation was not found between them because not only CA and RA but also some other factors, e.g. control skills of the subjects and self-willingness of the cyborg, might influence the control performance of such an integrated system.

The overall results of online experiments were given in Table 2. On average the subjects took 30.8±16.7 s and sent 12.7±8.5 turning commands included 7.8±6.5 left-turn commands and 4.9±3.5 right-turn commands to the cyborg to complete the control task in this study. As could be seen from Table 2 as well, the cyborg showed highly accurate response to the specific stimulation (1.5V, 50Hz, 50% duty cycle and 500ms monopole square pulse train) in most cases, the mean RA could reach by 89.5±15% (left-turn accuracy (84.6±12.7%); right-turn accuracy (92.0±9.5%)). At the same time, in the aspect of human, SSVEP acted as an efficient BCI solution, mean CA of SSVEP on three subjects in training session reached by 86.0±10.4%, indicating that both BCI and neruomodulation sub-system we used hold the characteristic of high accuracy and hence a robust information transfer pathway from one brain to another could be established.

As a solid performance-evaluating indicator for the system, the mean CPC value reached 0.616±0.169 and was obviously higher than the chance level (0.375) (*t* = 8.170, *P*<0.0001, One-Sample T-Test). Besides, experiments for control groups were also taken in this study. In control groups, three cyborgs walked along the S-shape track freely without control from any human subjects for 10 trials separately. We kept track of the related parameters during the process and the SR of all control groups were 0%. However, the mean SR for the online experiments achieved with this functional BTBS system reached 20% and it was significantly higher than that of the control groups (*t* = 3.464, *P* = 0.0085, Paired T-test). A video of a successful navigation was available for demonstration via the link (https://www.youtube.com/watch?v=k5t6WkTkJkA).

## Discussion

The present study demonstrated that human’s intention, extracted from the subject’s EEG, could be transmitted to partially dominate an insect’s brain. The human could successfully steer a cockroach to make turns using thoughts through SSVEP-based BCI and micro invasive neural stimulation.

The SSVEP could achieve a high CA (90.21%) by only using 4 channels on the scalp of visual cortex with a portable and wireless EEG device in the training phase (Fig 5). We also proposed an optimization algorithm for SSVEP, which greatly eliminated the random errors occurred during online control process, as a result, it significantly increased online classification accuracy by 8.08% from 83.34% to 91.42% with the removal of frequency-transient state signals. Generally, misclassification appeared during shifting period of a subject’s gaze in SSVEP. Therefore this phenomenon might be improved by adding a program to detect transition of the EEG signal on frequency before classification in further study, even though such increase of online classification accuracy was possibly at the sacrifice of SSVEP program’s fast-response performance. Besides, regarding the present SSVEP-based BCI, due to high brightness and repetitive stimulation, the user might suffer fatigue when using SSVEP for a long time, where fatigue was a generally feeling of tiredness, reduced concentration when performing a task [31]. The amplitude and SNR of the EEG signals were consequently affected by the fatigue [32, 33]. One possible solution to reduce fatigue of SSVEP was to use visual stimulation with high frequency or modulated amplitude [34, 35]. By implementing the possible improvements on SSVEP in further research, we can move current system a step closer to practical applications.

The other similar studies were compared with our work as well. Pais-Vieira *et al*. [1] succeeded in building up a BTBS for real-time sharing of sensorimotor information between two rats with intra-cortical microstimulation (ICMS), in which the decoder translated correctly 64.32% the motor information (two-mode) from the encoder under a time delay of 20.06 s (encoder) and 13.59 s (decoder), another experiment in the research demonstrated the decoder translated correctly encoder’s 62.34% tactile information (two-mode) and the time delay was 2.66 s (encoder) and 2.68 s (decoder) respectively. Recently, Rao *et al*. [3] accomplished a direct BTBS between humans, where the motor imaginary (two-mode) and TMS were applied to complete a task, the paired true positive rate (TPR) varied from 83.33% to 25.00% and paired false positive rate (FPR) varied from 0.00% to 37.50% in online experiments with a 2 s set-up time plus a 20 s visual countdown in each trial and a transmission duration of about 650 ms along the system. In this work, cyborgs (receiver) produced quick and accurate response (mean RA = 89.5%, left-turn accuracy (84.6%), right-turn accuracy (92.0%)) through applying the invasive technique of neuromodulation, where monopole square pulse trains were indirectly sent to the brains of the cyborg cockroach via antennas, and the time that a command sent from SSVEP to the completion of reaction from the cyborg was about 772 ms. In addition, the techniques we used in BCI (three-mode) segment in this study showed high decoding accuracy (CA = 86.0±10.4%) of sender’s intention as well and the average decoding time was 1.79 s (0.25 s minimum).

In the work of [22], Latif *et al*. tried to steer a cyborg cockroach to walk along an S-shape lap in two-directions manually and succeeded in achieving a success rate of around 10%. However, a successful online navigation of cyborg with human brain in this study was much more challenging, which required stable and continuous high level of accuracy in both “sender” and “receiver” sides, the experimental results demonstrated the BCI could successfully control the cyborg to walk along the S-shape track in all three groups, and the success rate ranged from 0.00% to 50.00% in 10-trial experiments, where the mean success rate (SR) was 20.00%, the value was not high enough, but significantly above the level in control group (0.00%) (*t* = 3.464, *P* = 0.0085). And the SR coincided with the roughly estimated value ((CA*RA)^*TC*^) based on the accuracy from both “sender” and “receiver” parts as there were 12.7 turning commands in average (Table 2) during each online trial. Additionally, the CPC, as an indicator of system performance, reached 0.616 (Table 2), while its chance level was 0.375. Above all, the results achieved through the system in present experiments were rather optimistic and instructive. In addition, the architecture of current system still had the potential of enabling developing multi-mode control cyborgs (more than three modes), and increasing the control modes might contribute to the improvement of SR in online control tasks and at the same time enable more precise online assignments as well, which would be designed and validated in further study.

It is meaningful to build up a BTBS between the human and the animal. Firstly, this all-chain-wireless system proves that it’s possible to built up an embryonic virtual brain-to-brain information transfer device with current technology. Besides, the idea also has the applicable value in reality, e.g. could be further used for detection in complex and dangerous environments. Most importantly, as a pioneer study of brain communication between human and animal, the successful implementation of BTBS expands the possible usage of both BCI and neuromodulation, furthermore, the alternative BCI and neuromodulation modalities (both invasive and non-invasive) may have huge potential of intersection and integration to create far-reaching usage in future. We have seen a mushroom growth of internet based on computers during recent years, and perhaps brain-to-brain network may be developed in the future just as the science-fiction films predict.
